# Transcriptomic exploration combined with experimental validation: uncovering the potential value of biomarkers related to ammonia-induced cell death in hepatic ischemia–reperfusion injury

**DOI:** 10.1186/s40001-025-03450-1

**Published:** 2025-11-26

**Authors:** Runyu Zhuang, Junhao Xiao, Benliang Mao, Yong Yan, Wei Yuan, Fan Wu, Bailin Wang

**Affiliations:** 1 Department of Hepatobiliary Surgery, Guangzhou Red Cross Hospital of Jinan University, Guangzhou, 510220 China; 2 Department of Hepatobiliary Surgery, Dong’guan KangHua Hospital, Dong’guan, 52300 China; 3 College of Clinical Medicine, Guizhou Medical University, Guiyang, 50004 China

**Keywords:** Hepatic ischemia–reperfusion injury, Ammonia-induced cell death, Diagnosis, Biomarkers

## Abstract

**Background:**

Hepatic ischemia–reperfusion injury (HIRI) represents the leading cause of postoperative liver dysfunction and failure. Ammonia-induced cell death (ACD), defined by lysosomal and mitochondrial disruption due to intracellular ammonia accumulation, appears to contribute to the pathogenesis of HIRI.

**Methods:**

Transcriptomic datasets GSE151648 and GSE12720 were retrieved from the Gene Expression Omnibus (GEO), and 467 ACD-related genes were compiled from published reports. Differential expression analysis combined with Weighted Gene Co-expression Network Analysis (WGCNA) was applied to identify candidate genes and assess their functional relevance. Biomarkers closely associated with HIRI were subsequently determined through protein–protein interaction (PPI) network construction, machine learning approaches, and expression validation. A nomogram was then established based on these biomarkers, followed by Gene Set Enrichment Analysis (GSEA), immune infiltration profiling, and network prediction. Furthermore, single-cell analysis was employed to investigate the expression of biomarkers across different cell types. Finally, liver tissues from HIRI mouse models were examined to confirm biomarker expression.

**Results:**

A total of 586 differentially expressed genes intersected with 762 key module genes, yielding 39 candidates primarily enriched in inflammatory signaling pathways. Among these, LCP1, SLC16A3, and RGS2 emerged as biomarkers, each markedly upregulated in HIRI samples. The biomarker-based nomogram demonstrated robust diagnostic accuracy. Enrichment analyses indicated that the biomarkers were closely related to immune and metabolic pathways. Consistently, immune cell infiltration and immune functions were elevated in HIRI samples and correlated significantly with biomarker expression. Concurrently, single-cell analysis revealed that all three biomarkers were expressed within mononuclear phagocytes, with their expression levels exhibiting significant differences between the HIRI group and the control group. Moreover, multiple miRNAs and lncRNAs were predicted to interact with the identified biomarkers. Validation in HIRI mouse liver tissues confirmed consistency with transcriptomic findings.

**Conclusion:**

LCP1, SLC16A3, and RGS2 have been identified as biomarkers of HIRI. The study advances understanding of ACD-related genes signatures in HIRI and provides a foundation for future mechanistic research and therapeutic development.

**Supplementary Information:**

The online version contains supplementary material available at 10.1186/s40001-025-03450-1.

## Introduction

Ischemia–reperfusion injury (IRI) describes the paradoxical condition in which restoration of blood supply to ischemic tissues intensifies cellular injury rather than attenuating it, culminating in metabolic dysfunction and structural disruption [1]. Hepatic ischemia–reperfusion injury (HIRI), frequently encountered during liver transplantation and hepatectomy, constitutes a leading contributor to postoperative hepatic impairment and graft failure [2]. This process can provoke abrupt functional decline and heighten the likelihood of transplant rejection [3]. The pathophysiological cascade of HIRI unfolds in two sequential phases—ischemia and reperfusion—characterized, respectively, by hepatocellular hypoxia and subsequent bursts of pro-inflammatory cytokines and reactive oxygen species (ROS) [4]. Current therapeutic strategies, including surgical manipulation, pharmacological regimens, and novel modalities, such as autophagy regulation or photothermal treatment, offer only partial benefit, with overall clinical outcomes remaining unsatisfactory [5, 6]. From a diagnostic standpoint, early identification and continuous assessment of HIRI are hindered by non-specific clinical manifestations, absence of reliable biomarkers, and the intricate interplay of its pathological mechanisms [7]. Consequently, the establishment of dependable biomarkers is indispensable for elucidating disease mechanisms, refining therapeutic approaches, and advancing the development of targeted interventions.

Ammonia is a cellular toxin, and elevated blood concentrations can impair cellular functions [8]. Ammonia-induced cell death (ACD) has recently been defined as a distinct death modality initiated by intracellular ammonia accumulation, predominantly driven by lysosomal and mitochondrial injury [8, 9]. Hallmarks of ACD include lysosomal alkalinization, mitochondrial swelling, and defective autophagic flux [9]. Entry of ammonia into lysosomes occurs through the Rh Family C Glycoprotein (RHCG) transporter, where its protonation to NH₄⁺ elevates lysosomal pH and disrupts lysosomal integrity. Subsequent reflux of ammonia into mitochondria provokes structural and functional damage, reflected in diminished membrane potential, swelling, and bioenergetic failure [8, 10]. The liver serves as the primary site for ammonia metabolism, with hepatocytes being the target cells of ammonia toxicity. Hepatocyte damage leads to abnormal ammonia metabolism, while hyperammonemia caused by excessive ammonia accumulation disrupts normal metabolic processes in hepatocytes [11]. Previous studies have revealed that mitochondrial structural abnormalities and functional impairments compromise cellular homeostasis, playing a pivotal role in the pathogenesis of IRI [12]. Consequently, ammonia metabolic disorders induced by hepatic ischemia–reperfusion injury lead to ammonia accumulation. The impaired metabolic function of hepatocytes under high-ammonia conditions further exacerbates hepatic dysfunction. ACD characterized by lysosomal and mitochondrial damage may play a significant role in this process. Although various forms of cell death have been associated with HIRI, such as pyroptosis [13] and ferroptosis [14], systematic research on the biological role of ACD-related genes (ACDRGs) in hepatic ischemia–reperfusion injury (HRI) remains relatively limited.

The objective of this study was to identify biomarkers of ACD in HIRI by integrating multi-level bioinformatic approaches. Differential expression analysis and weighted gene co-expression network analysis (WGCNA) were first applied to screen candidate genes, followed by functional annotation. Subsequent integration of machine learning algorithms, protein–protein interaction (PPI) network analysis, and expression validation enabled the recognition of HIRI-associated biomarkers. On this basis, a nomogram was established to predict clinical outcomes, and mechanistic insights were pursued through gene set enrichment analysis (GSEA) and immune pathway analysis. Validation of biomarker expression was further performed using a HIRI mouse model. Elucidating the relationship between ACD and HIRI is expected to advance understanding of HIRI pathogenesis and clarify the mechanisms underlying this newly defined mode of cell death, while also providing a framework for future mechanistic studies and therapeutic strategies.

## Materials and methods

### Data source

The training dataset GSE151648 and the validation dataset GSE12720 were obtained from the Gene Expression Omnibus (GEO) database (https://www.ncbi.nlm.nih.gov/geo/). GSE151648 [15], generated on the GPL21290 platform through high-throughput sequencing, comprised 46 hepatic tissue samples from HIRI cases (IRI⁺) and 34 control hepatic tissue samples (IRI⁻). GSE12720 [16], derived from the GPL570 platform, contained 21 hepatic tissue samples with HIRI and 21 matched control samples. GSE171539 [17] is a single-cell dataset generated via high-throughput sequencing on the GPL20795 platform, comprising one pre-procurement sample and one post-reperfusion sample. Additionally, 467 ACDRGs were extracted from the MSigDB database (https://www.gsea-msigdb.org/gsea/msigdb, access time: 2025-03-07), including genes linked to RHCG, GLS, autophagy, macroautophagy, ammonia, ammonium, lysosomal pH regulation, and effector T cells [8] (Additional file 1).

### Weighted gene co-expression network analysis (WGCNA)

In the training dataset, the “rstatix” package (v0.7.2) was used to conduct Wilcoxon tests comparing ACDRG expression levels between HIRI and control samples (*P* < 0.05). Differentially expressed ACDRGs were then subjected to ssGSEA using the “GSVA” package (v1.53.28) [18], and group differences in ssGSEA scores were reassessed with the Wilcoxon test (*P* < 0.05). Visualization of statistical outcomes was generated with the “ggplot2” package (v3.5.1) [19].

Sample clustering was performed with the hclust function in the “WGCNA” package [20], and only outlier-free samples were retained. For network construction, select the unsigned type (TOMType = “unsigned”), meaning only the absolute correlations of gene expression values are considered. Network topology analysis was carried out with the pickSoftThreshold function, and an optimal soft-thresholding power was chosen to construct a scale-free network with a fitting index threshold of *R*^2^ > 0.85. Subsequently, the dynamic tree cutting algorithm was employed to partition the genes into modules, with a minimum module size of 100 genes and a module merging threshold of 0.25 set to consolidate highly similar modules. Pearson correlation analysis was applied to evaluate associations between ssGSEA scores of ACDRGs and module eigengenes, and modules exhibiting significant correlations (|cor|> 0.3, *P* < 0.05) were designated as key modules. Genes within these modules were identified as key module genes.

### Identification of differentially expressed genes (DEGs)

In the training dataset, DEGs between HIRI and control samples were identified using the “DESeq2” package (v1.40.2) [21], with thresholds of |log₂FC|> 0.5 and *P* < 0.05. Global expression profiles were visualized through a volcano plot generated by the “ggplot2” package (v3.5.1), with the top five DEGs annotated according to descending |log₂FC| values. A heatmap illustrating the expression distribution of the ten most dysregulated DEGs, ranked by descending |log₂FC| values, was constructed using the “ComplexHeatmap” package (v2.16.0) [22].

### Acquisition and functional exploration of candidate genes

Candidate genes were determined as the overlap between DEGs and key module genes using the “ggvenn” package (v0.1.10) [23]. Their potential biological roles and associated signaling pathways were examined by Gene Ontology (GO) and Kyoto Encyclopedia of Genes and Genomes (KEGG) enrichment analyses. GO categories included biological process (BP), cellular component (CC), and molecular function (MF). Enrichment analysis was conducted with the “clusterProfiler” package (v4.15.0.003) [24], adopting a significance threshold of *P* < 0.05. Results were prioritized by descending gene count and displayed accordingly. To investigate molecular interactions, a PPI network of candidate genes was generated through the STRING database (http://string-db.org) with a minimum confidence score > 0.15. Node connectivity within the PPI network was assessed using the Closeness and Clustering Coefficient algorithms available in the CytoHubba plugin of Cytoscape (v3.10.2) [25]. The top 20 genes ranked by each algorithm were extracted, and their intersection was further defined through the “ggvenn” package to identify key candidate genes.

### Machine learning

Within the training set, candidate genes were refined through support vector machine–recursive feature elimination (SVM-RFE) and random forest algorithms. The “e1071” package (v1.7–16) [26] was applied to implement SVM-RFE, and five-fold cross-validation was performed to identify the gene subset associated with the lowest error rate or the highest predictive accuracy. In parallel, random forest analysis was carried out using the “randomForest” package (v4.7–1.2) [27] under the parameters ntree = 300 and mtry = 2. Genes were prioritized according to the MeanDecreaseGini index, and the top 10 were retained as random forest genes. To integrate results from both approaches, the “ggvenn” package (v0.1.10) was used to compute the overlap between the SVM-RFE and random forest outputs, and the intersecting genes were designated as candidate biomarkers.

### Expression validation

Expression levels of candidate biomarkers were compared between HIRI and control samples across training and validation cohorts using the Wilcoxon test (*P* < 0.05). Genes exhibiting statistically significant differences along with consistent expression patterns in both cohorts were defined as final biomarkers for downstream analyses.

### Construction of nomogram

In the training set GSE151648 and validation set GSE12720, a nomogram integrating the selected biomarkers was generated with the “rms” package (v6.8–1) [28] to estimate the probability of HIRI occurrence. Its predictive capacity was examined by constructing a calibration curve with the same package, a decision curve analysis (DCA) with the “ggDCA” package (v1.1), and a receiver operating characteristic (ROC) curve using the “pROC” package (v1.18.5) [29], where the area under the curve (AUC) ranged between 0.7 and 1.

### Construction of networks

MicroRNAs (miRNAs) associated with the identified biomarkers were retrieved from the ENCORI database (https://rnasysu.com/encori/). Long non-coding RNAs (lncRNAs) targeting these miRNAs were subsequently predicted through the StarBase database (http://starbase.sysu.edu.cn/). Cytoscape software (v3.10.2) was then applied to establish the lncRNA–miRNA–mRNA regulatory network. Furthermore, the identified biomarkers were uploaded to the GeneMANIA platform (http://genemania.org/) to infer functionally related genes and shared signaling pathways.

### Gene set enrichment analysis (GSEA)

Within the training cohort, Spearman correlation coefficients were calculated between each biomarker and all other genes using the “psych” package (v2.4.6.26) [30], and the genes were ranked in descending order of correlation values. GSEA was subsequently performed on this ranked expression matrix using the “clusterProfiler” package (v4.15.0.003), applying thresholds of adjust *P* < 0.05, false discovery rate (FDR) < 0.25, and | normalized enrichment score (NES)|> 1. The top five pathways were reported after sorting by ascending p-values. The background reference gene set “c2.cp.kegg_legacy.v2025.1.Hs.symbols.gmtt” was obtained from the MSigDB database.

#### Immune infiltration analysis

Immune cell subsets and immune functions were employed as evaluation parameters. In the training set, the ssGSEA algorithm implemented in the “GSVA” package (v1.53.28) was applied to quantify infiltration levels of 28 immune cell types [31] and activity scores of 13 immune functions [32] in each sample. Concurrently, to further enhance the robustness of cell-type inference, we employed the xCell algorithm for immune cell infiltration analysis. This tool estimates the relative abundance of 64 distinct immune cell types based on gene signatures [33]. Group comparisons between HIRI and control samples were conducted using the Wilcoxon test (*P* < 0.05). Immune cell subsets and immune functions showing significant variation were further examined for correlations with identified biomarkers through Spearman analysis using the “psych” package (v2.4.6.26) (|cor|> 0.3, *P* < 0.05).

#### Drug prediction and molecular docking

Candidate drugs targeting the identified biomarkers were predicted using the DGIdb database (https://www.dgidb.org/), and the drug–biomarker interaction network was generated with Cytoscape software (v3.10.2). Three-dimensional drug structures were retrieved from PubChem (https://pubchem.ncbi.nlm.nih.gov/), while homologous protein structures and UniProt Entry numbers of biomarkers were obtained from the UniProt database (https://www.uniprot.org/).

#### Single-cell analysis

To investigate the cell-specific expression patterns of ammonia-related biomarkers in HIRI, we performed single-cell analysis using the R package Seurat (v 5.30) [17]. Initially, quality control was performed on the single-cell dataset GSE171539 using the PercentageFeatureSet function, applying screening criteria of nCount_RNA ≤ 12,000, 3000 > nFeature_RNA > 600, and percent.mt < 10% to exclude low-quality cells and polyploidy. Subsequently, data were integrated and samples merged using IntegrateData, normalized via NormalizeData, and the top 2000 highly variable genes were selected using FindVariableFeatures. The 10 most significantly differentially expressed genes (*P* < 0.05) were visualized using LabelPoints. Subsequently, to further confirm the cell populations to which clustered cells belonged, ScaleData was employed for normalization. runPCA was then used to perform principal component dimensionality reduction on highly variable genes. The significance of the top 50 principal components was calculated via the Jackstraw permutation test, while the ElbowPlot determined the critical dimension. Unsupervised clustering was then performed using FindNeighbors and FindClusters, with RunUMAP (resolution = 0.2) visualizing the results. Manual annotation of cell clusters was conducted using signature genes from literature references [17, 34], with annotated results again visualized via UMAP. Finally, biomarker expression levels within cells were detected, with Wilcoxon tests comparing expression levels between normal and diseased states (*P* < 0.05).

#### HIRI mouse model establishment and reverse transcription-quantitative polymerase chain reaction (RT-qPCR)

Seven-week-old male C57BL/6J mice (Guangzhou Yancheng Biotechnology Co., Ltd., China) were subjected to total warm hepatic ischemia–reperfusion procedures. Twenty mice were purchased and housed at room temperature (23 ±  °C) with a 12-h light–dark cycle. They were provided free access to food and water for one week to ensure consistent baseline conditions across all subjects. The mice were then randomly divided into two groups: the Ischemia–Reperfusion Model Group (HIRI group) and the Control Group, with 10 mice in each group. To induce approximately 70% hepatic ischemia, the left and middle hepatic vascular trunks were clamped using vascular clips, causing ischemia in the left and middle hepatic lobes. Within minutes, these lobes turned clay-gray or pale white, while the right lobe remained bright red. After confirming complete blood flow occlusion, the abdominal retractor was removed, the gastrointestinal tract was repositioned into the abdominal cavity, and the incision was temporarily closed. Following a 30-min ischemic period, the hepatic hilum was re-exposed. Reperfusion was confirmed by the return of normal color in the affected lobes, indicating successful establishment of the HIRI model. Six hours post-reperfusion, mice were euthanized to harvest serum and liver tissue samples. After blood sample collection, serum alanine aminotransferase (ALT), aspartate aminotransferase (AST), and lactate dehydrogenase (LDH) levels were measured using Sigma-Aldrich analysis kit. For liver histopathological analysis, the left hepatic lobe was uniformly dissected, fixed in 4% paraformaldehyde solution for 24 h, and then subjected to sequential processing including dehydration, embedding, paraffin sectioning, and H&E staining performed in accordance with the manufacturer's instructions. Residual liver tissues were fixed in tissue fixative and stored at −80°C for subsequent quantitative real-time polymerase chain reaction (qPCR) assays. The control group underwent laparotomy alone without vascular occlusion, with all other experimental procedures being identical to those applied to the HIRI group.

Ten liver tissue samples from each group were harvested. Tissues were lysed with TRIzol reagent (Nanjing Vazyme Biotech Co., Ltd., China), and total RNA was extracted and quantified. cDNA synthesis was performed using the Hifair^®^ III 1 st Strand cDNA Synthesis SuperMix for qPCR kit in accordance with the manufacturer’s protocol, with reverse transcription carried out on an S1000™ Thermal Cycler (BIO-RAD, USA) (Additional file 2). Specific primers for target biomarkers were designed (Table 1), and amplification was conducted for 40 cycles on a CFX Connect Real-Time PCR System (BIO-RAD, USA) (Additional file 3). GAPDH was employed as the housekeeping gene, and relative gene expression levels were calculated using the 2^–ΔΔCt^ method. All samples underwent triplicate technical replicates. Furthermore, given the small sample size, this study did not perform normality tests on PCR data. Instead, non-parametric methods (Mann–Whitney *U* test) were employed for intergroup comparisons to ensure robust statistical results (*P* < 0.05). Data visualization was completed using GraphPad Prism 10 software.

**Table 1:** RT-qPCR primer sequence list

primer	sequences
LCP1 F	TGTGCCAGACACGATTGACG
LCP1 R	TCGGCCCCTATATTAACCACG
SLC16A3 F	GCTGGCGGTAACAGGTGAA
SLC16A3 R	GGAAGGCGTAGGAGAAACCC
RGS2 F	GTGCAAGGGTGTTGACGTTC
RGS2 R	ACAGACGCTGGTTCTACAGC
M-GAPDH F	TGTGTCCGTCGTGGATCTGA
M-GAPDH R	GAGTTGCTGTTGAAGTCGCA

#### Statistical analysis

Statistical analyses of the bioinformatics data were conducted using R software (v4.2.2). Intergroup comparisons were assessed with the Wilcoxon test, applying a significance threshold of *P* < 0.05. Network visualizations were generated in Cytoscape software (v3.10.2). For RT-qPCR validation, group differences in biomarker expression were determined using the Mann–Whitney U test, with statistical significance defined as *P* < 0.05.

## Results

### A total of 762 key module genes were acquired

**Fig. 1:**
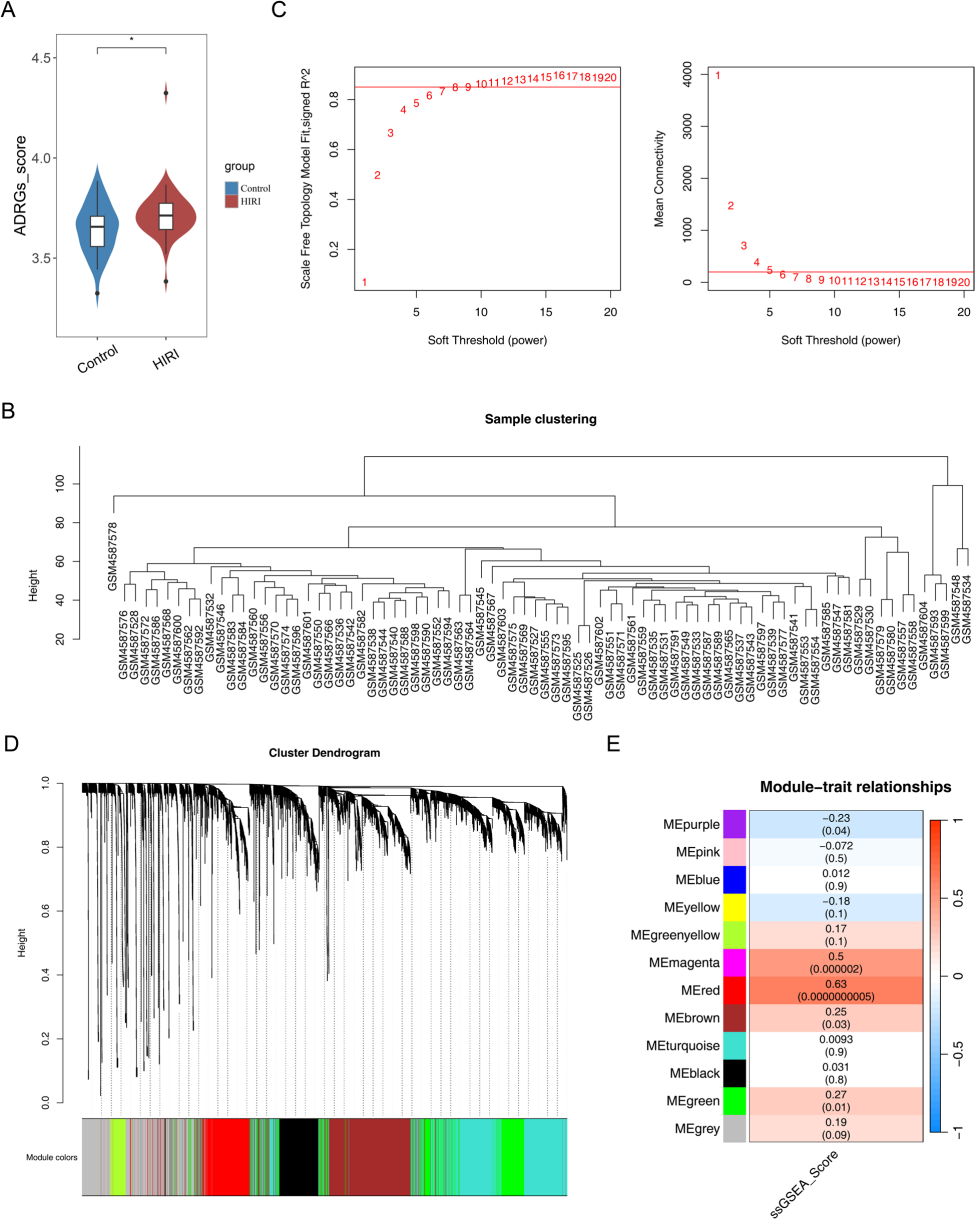
A total of 762 key module genes were acquired.** A** ACDRGs scores for the HIRI sample and control sample. **B** Sample clustering and outlier detection. **C** Screening of soft thresholds. The left panel shows the scale-free fitting index for different soft thresholds, while the right panel displays network connectivity for varying soft thresholds. **D** Clustering dendrogram for different modules. **E** Heatmap illustrating the correlation between modules and ACDRGs scores

Analysis revealed that ssGSEA scores of differentially expressed ACDRGs were markedly higher in HIRI samples (*P* < 0.05) (Fig. 1A). Based on ssGSEA scores of ACDRGs, WGCNA was subsequently applied. After exclusion of outliers, a soft threshold of 9 was selected to establish a scale-free topology, with network connectivity approaching zero (Fig. 1B–C). The constructed network divided genes in the training set into 12 modules, including a gray module representing unclustered genes (Fig. 1D). Among the modules, MEred and MEmagenta showed strong correlations with ssGSEA scores of ADRGs, with correlation coefficients of 0.63 and 0.50, respectively (*P* < 0.0001) (Fig. 1E). Genes from these two modules were integrated, yielding a total of 762 key module genes (Additional file 4).

### The candidate genes were concerned with multiple aspects of functions

**Fig. 2:**
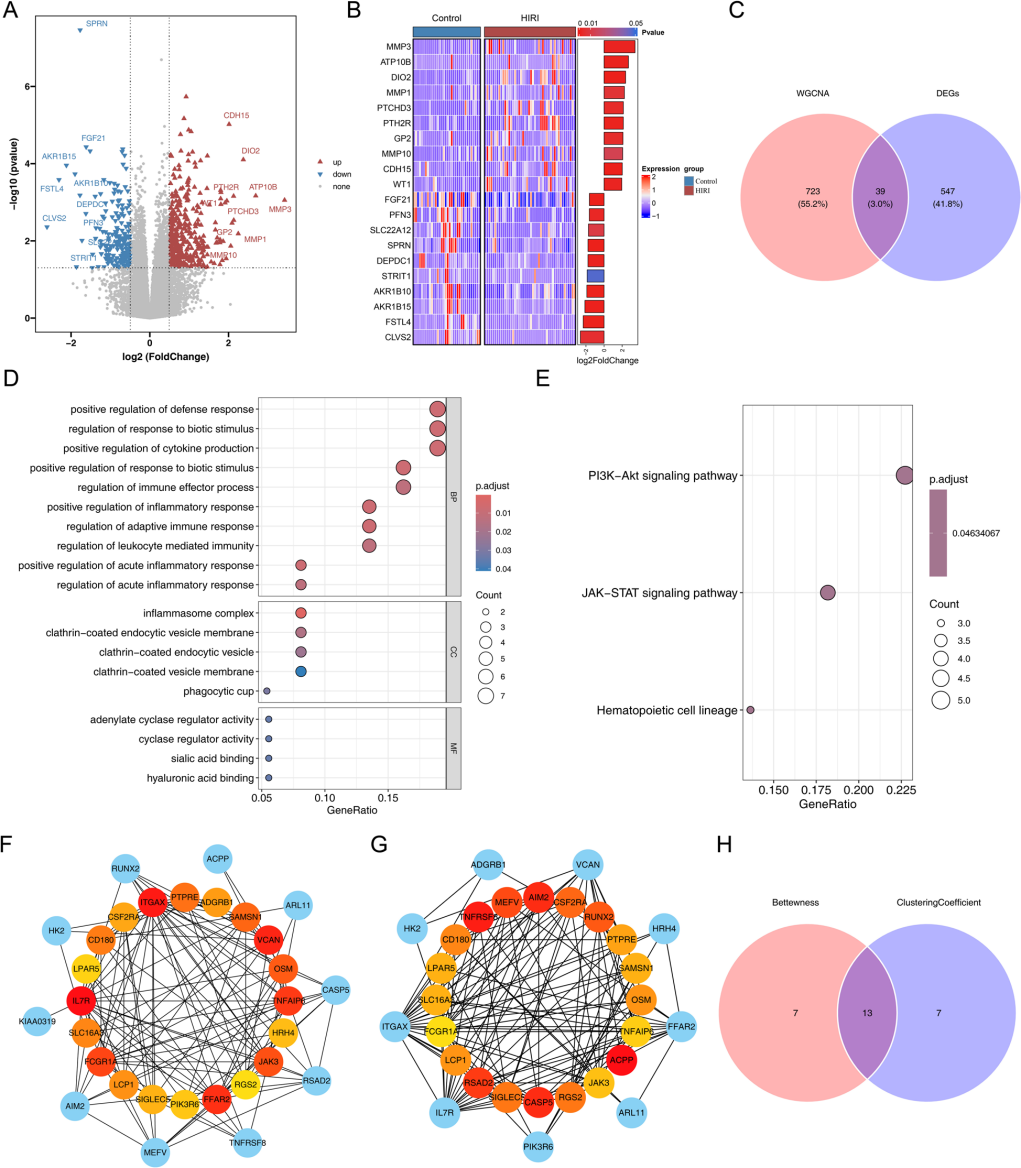
Acquisition and functional exploration of candidate genes. **A** Volcano plot of DEGs between HIRI and control samples. **B** Heatmap of DEGs between HIRI samples and control samples. **C** Candidate genes obtained by the intersection of DEGs and key module genes. **D** The top 10 biological processes with the highest enrichment of candidate genes. **E** Obtained from KEGG pathway analysis of pathways involving candidate genes. **F** PPI network of candidate genes (Betweenness algorithm). **G** PPI network of candidate genes (Clustering Coefficient algorithm). **H** Venn diagram analysis of key candidate genes. Overlap between the top 20 gene lists derived from the Betweenness and Clustering Coefficient algorithms

Within the training set, 586 DEGs were detected in HIRI samples, comprising 400 upregulated (log₂FC > 0.5) and 186 downregulated genes (log₂FC < − 0.5) (Fig. 2A–B**, **Additional file 5). Intersecting the 762 key module genes with these DEGs resulted in 39 candidate genes (Fig. 2C).

Gene Ontology-Biological Process (GO-BP) enrichment analysis indicated that the candidate genes clustered into 25 biological processes, with major involvement in “positive regulation of defense response,” “regulation of response to biotic stimulus,” and “positive regulation of cytokine production.” GO-CC terms highlighted associations with five structural components, including the “inflammasome complex” and “clathrin-coated endocytic vesicle membrane,” while GO-MF analysis pointed to four functional terms such as “adenylate cyclase regulator activity” and “cyclase regulator activity.” The top 10 enriched GO-BP categories together with all GO-CC and GO-MF results are summarized in Fig. 2D and Additional file 6.

KEGG pathway analysis further demonstrated predominant enrichment in the “JAK-STAT signaling pathway” and “PI3K-Akt signaling pathway” (Fig. 2E). In the PPI network, 13 candidate genes emerged as key nodes, representing the overlap between the top 20 ranked by the Betweenness and Clustering Coefficient algorithms (Fig. 2F–H).

### LCP1, SLC16A3, and RGS2 were identified as biomarkers

**Fig. 3:**
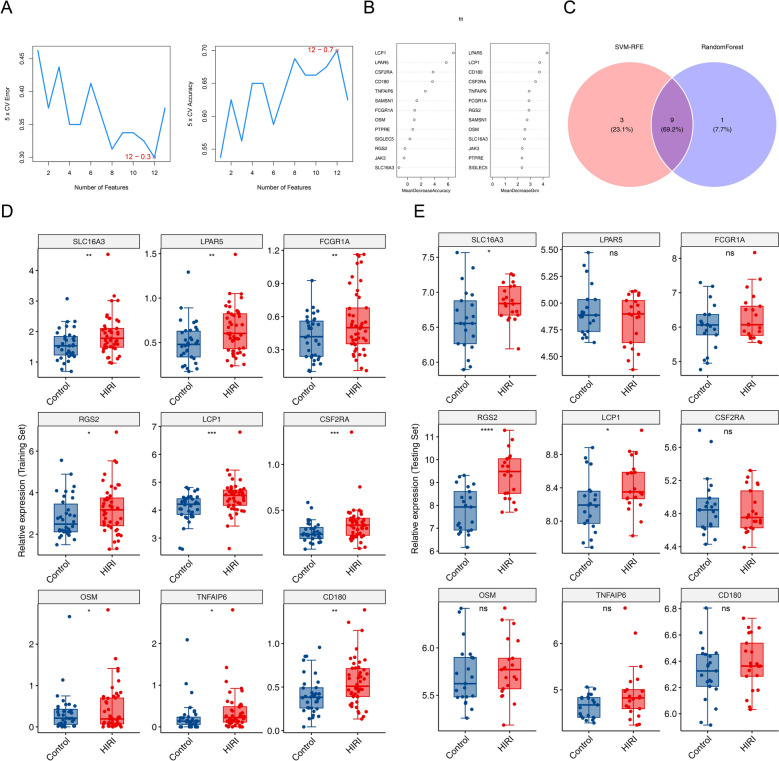
LCP1, SLC16A3, and RGS2 were identified as biomarkers. **A** In the SVM-RFE algorithm, the accuracy of five-fold cross-validation (CV) and the error of five-fold CV in the SVM-RFE algorithm. **B** Random forest model diagram. **C** Venn diagram of intersection genes between random forest and SVM-RFE algorithms. **D** Expression profiles of candidate biomarkers in the control group and HIRI in the training set. **E** Expression profiles of candidate biomarkers in the control group and HIRI in the validation set

The SVM-RFE model reached optimal performance at its minimum error rate or maximum accuracy, yielding 12 candidate genes: *JAK3, SLC16A3*, *LPAR5, FCGR1A, RGS2, SIGLEC5, PTPRE, LCP1, CSF2RA, OSM, TNFAIP6*, and *CD180* (Fig. 3A). In parallel, the random forest model ranked the top 10 genes by MeanDecreaseGini as *LPAR5, LCP1, CD180, CSF2RA, TNFAIP6, FCGR1A, RGS2, SAMSN1, OSM*, and *SLC16A3* (Fig. 3B). Nine genes were shared across both algorithms (Fig. 3C). Among these, *LCP1*, *SLC16A3*, and *RGS2* were significantly upregulated in both the training and validation sets (*P* < 0.05). Therefore, *LCP1*, *SLC16A3*, and *RGS2* were identified as biomarkers (Fig. 3D–E).

### The nomogram was able to predict the risk of HIRI in samples

**Fig. 4:**
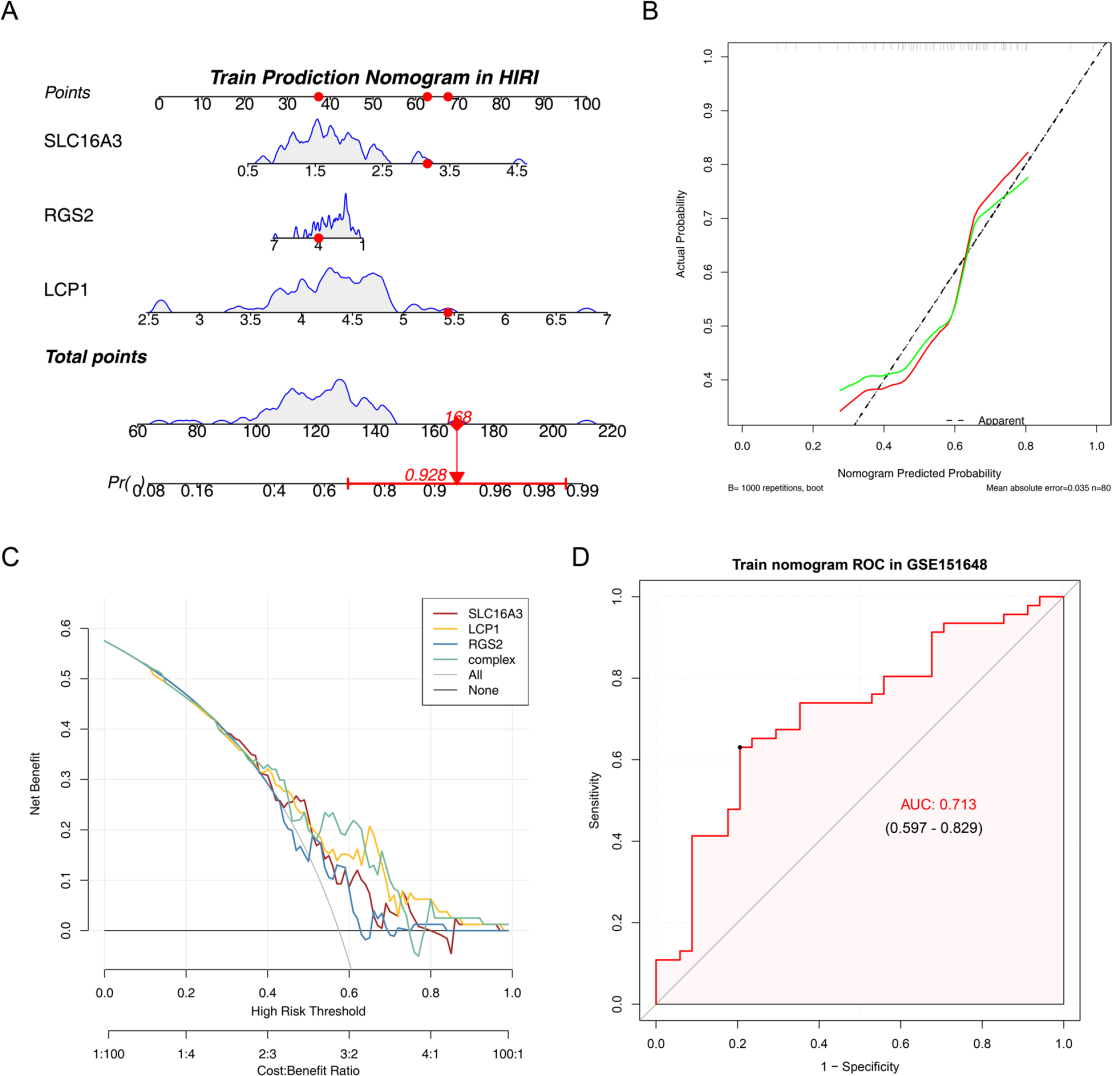
The nomogram was able to predict the risk of HIRI in samples (training set GSE151648). **A** Nomogram constructed to assess diagnostic value of biomarkers. **B** Calibration curve evaluating agreement between predicted and observed outcomes. **C** DCA curve reflecting clinical utility of the model. **D** ROC curve illustrating predictive performance of the nomogram

Based on the biomarkers identified within the training dataset GSE151648, we constructed a nomogram for this dataset **(**Fig. 4A). *LCP1* exerted the greatest influence, indicated by its longest scale line. The red dot illustrates the biomarker scores of a representative training sample, corresponding to a total of 168 points and a predicted HIRI probability of 92.8%. The calibration curve revealed close concordance between predicted and observed outcomes (Fig. 4B). DCA demonstrated superior net clinical benefit of the nomogram relative to the "All" and "None" approaches (Fig. 4C). Receiver operating characteristic (ROC) analysis further validated the model, with an AUC of 0.713 (AUC > 0.7) (Fig. 4D). Collectively, the three evaluation curves confirmed that the nomogram provided a certain degree of diagnostic accuracy. Moreover, in the validation set GSE12720, the nomogram constructed based on the aforementioned biomarkers similarly exhibited robust diagnostic potential (Additional file 7A–D).

### The biomarkers were concerned with different regulatory relationships

**Fig. 5:**
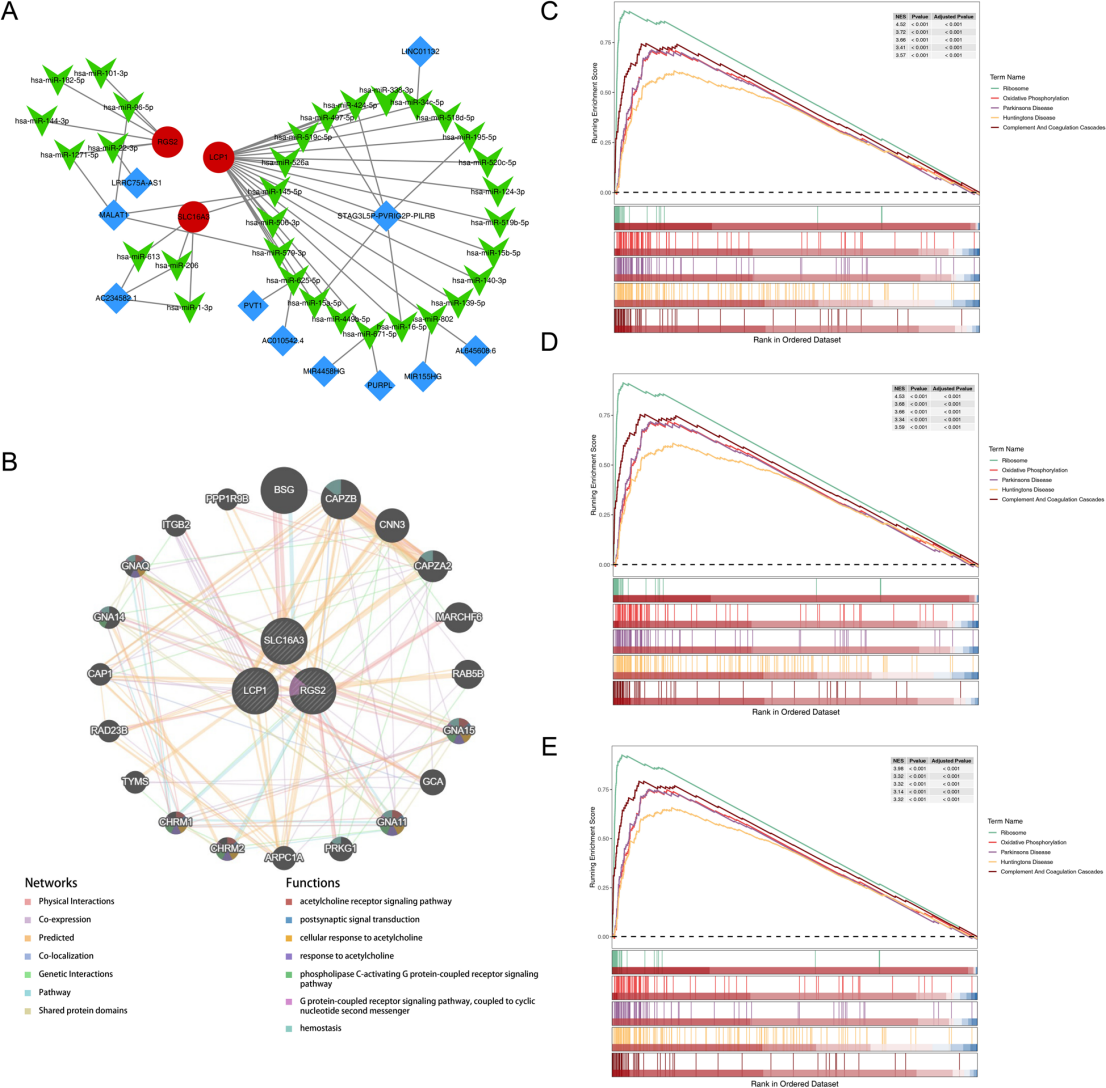
The biomarkers were associated with immunity and metabolism. **A** lncRNA–miRNA–mRNA network. Red and blue rectangles represent lncRNAs, red circles represent mRNAs, and green chevrons represent miRNAs. **B** GeneMANIA network. **C**–**E** GSEA results of the LCP (**C**) RGS2 (**D**) SLC16A3 (**E**), displaying the top 5 most significant pathways

LCP1, SLC16A3, and RGS2 were predicted to be regulated by 23, 4, and 6 miRNAs, respectively. For LCP1, eight lncRNAs were identified as upstream regulators of its targeting miRNAs, generating axes such as STAG3L5P–PVRIG2P–PILRB–*hsa-miR-15a-5p*–LCP1. RGS2-related axes included MALAT1–*hsa-miR-1271-5p*–RGS2, derived from two lncRNAs linked to RGS2-targeting miRNAs. In contrast, SLC16A3-targeting miRNAs converged on AC234582.1, exemplified by the axis AC234582.1–*hsa-miR-613*–SLC16A3 (Fig. 5A). To further define functional associations, a GGI network was constructed using the three biomarkers and 20 functionally similar genes (e.g., *BSG*, *CAPZB*). Within this network, RGS2 exhibited strong association with the “phospholipase C-activating G protein-coupled receptor signaling pathway” in combination with *GNA15, GNA11*, and *CHRM1* (Fig. 5B). Collectively, the biomarkers appear embedded in multilayered regulatory circuits that may constitute mechanistic drivers of AD in the context of HIRI.

### The biomarkers were associated with immunity and metabolism

KEGG enrichment analysis revealed predominant involvement of the biomarkers in immune-related and metabolic pathways. The genes LCP1, RGS2, and SLC16A3 were all jointly involved in core pathways such as “ribosome,” “oxidative phosphorylation,” and “complement and coagulation cascade,” with a total of 131, 132, and 127 associated pathways respectively (Additional file 8–10) The five most significantly enriched pathways for each biomarker, ranked by *P*-value, are depicted in Fig. 5C–E.

### The immune activity is increased in HIRI samples

**Fig. 6:**
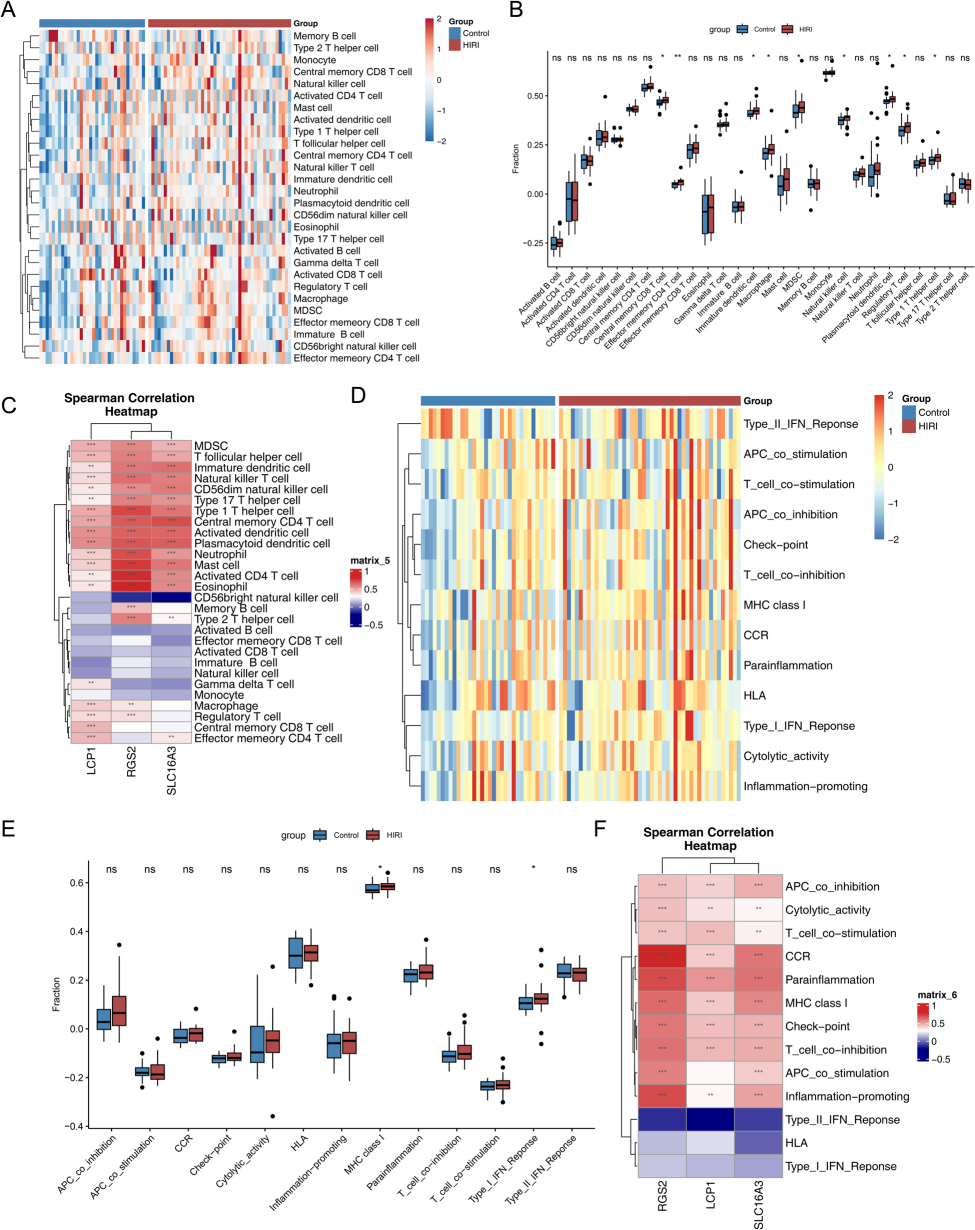
The immune activity is increased in HIRI samples. **A** Heatmap depicting immune scores of 28 immune cell types in HIRI and control groups. **B** Box plot comparing immune scores of 28 immune cell types across groups. **C** Heatmap of correlations between differential immune cells and biomarkers. **D** Heatmap of immune scores for 10 immune functions, with red indicating activation and blue indicating suppression. **E** Box plot of immune function scores across groups. **F** Heatmap of correlations between differential immune functions and biomarkers. **G** Drug–biomarker interaction network, with blue nodes representing drugs and pink nodes representing biomarkers. *Ns* not significant; *p < 0.05, **p < 0.01, ***p < 0.001

Analysis of 28 immune cell subsets revealed significantly increased infiltration of 9 cell types in HIRI samples, including immature dendritic cells, macrophages, myeloid-derived suppressor cells (MDSCs), natural killer cells, and plasmacytoid dendritic cells (*P* < 0.05) (Fig. 6A–B). The biomarkers **RGS2** and **SLC16A3** demonstrated positive correlations (cor > 0.3, *P* < 0.0001) with nearly half of the immune cell subsets, notably MDSCs, natural killer T cells, and type 1 T helper cells (Fig. 6C). In terms of immune functional activity, MHC class I activity and the type I IFN response were markedly elevated in HIRI samples (*P* < 0.05) (Fig. 6D–E). Moreover, the biomarkers displayed strong positive associations with multiple immune functions, particularly CCR and MHC class I (Fig. 6F). Collectively, the results indicate a close interconnection among immune infiltration, biomarker expression, and immune function alterations in HIRI, suggesting that ACD-associated gene signatures may contribute to immune regulation during its pathogenesis.

Among the 64 immune cell types analyzed, 12 cell types exhibited significantly different infiltration levels between HIRI and normal samples, including class-switched memory B cells, endothelial cells, and memory B cells (Additional file 11A-B). Further correlation analysis revealed that biomarker LCP1 expression exhibited a significant positive correlation with conventional dendritic cell (cDC) infiltration levels (cor > 0.3, *P* < 0.0001). Both biomarkers RGS2 and SLC16A3 showed positive correlations with neutrophil levels (cor > 0.3, *P* < 0.0001), but negative correlations with plasma cell levels (cor < − 0.3, *P* < 0.0001) (Additional file 11 C).

### Potentially effective medications

The DGIdb database identified SLC16A3 as a target of streptozocin and RGS2 as a target of haloperidol decanoate, while no drug interactions were detected for LCP1.

### The biomarkers are all expressed in mononuclear phagocytes.

**Fig. 7:**
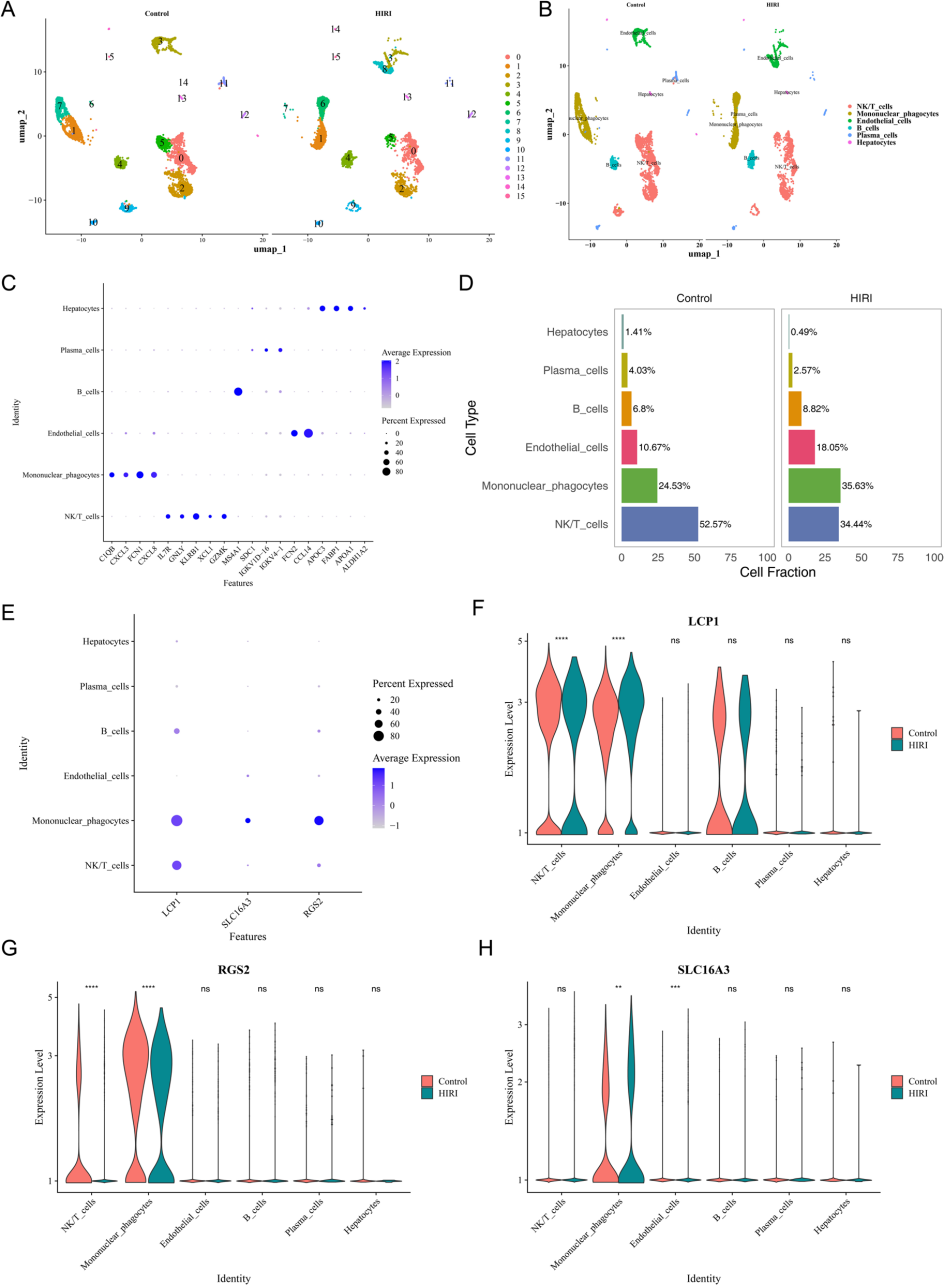
Cell annotation and biomarker expression**.** **A** Clustering analysis results. **B** Cell annotation results. **C** Expression status of marker genes in annotated cells. **D** Proportion of cells in the normal group and HIRI group. **E** Bubble plot illustrating biomarker expression across cell types. **F**–**G** Violin plots showing biomarker expression across cell types

Following quality control of the single-cell dataset GSE171539, a total of 7736 cells and 19,445 genes were retained (Additional file 12A-B). Cluster analysis partitioned all cells into 16 subpopulations, further annotated as six HIRI-associated cell types: mononuclear phagocytes, NK/T cells, B cells, plasma cells, endothelial cells, and hepatocytes (Additional file 12C-E**, **Fig. 7A–C). Within the HIRI model, mononuclear phagocytes constituted a higher proportion than in controls, emerging as the predominant cell type; conversely, NK/T cells exhibited a declining trend (Fig. 7D). Further investigation revealed that three biomarkers associated with ammonia-induced cell death were expressed in mononuclear phagocytes, with significant differences in expression levels between the HIRI and control groups. Notably, LCP1 and RGS2 also exhibited significant intergroup expression variations in NK/T cells. Notably, no significant differences in the expression of these three biomarkers were observed between the HIRI group and the control group in hepatocytes (Fig. 7E–H). This suggested that HIRI may promoted hepatic inflammation and injury by activating the ammonia-induced cell death pathway in immune cells such as mononuclear phagocytes.

#### Successful establishment of the HIRI mice model and qPCR results are consistent with expectations.

**Fig. 8:**
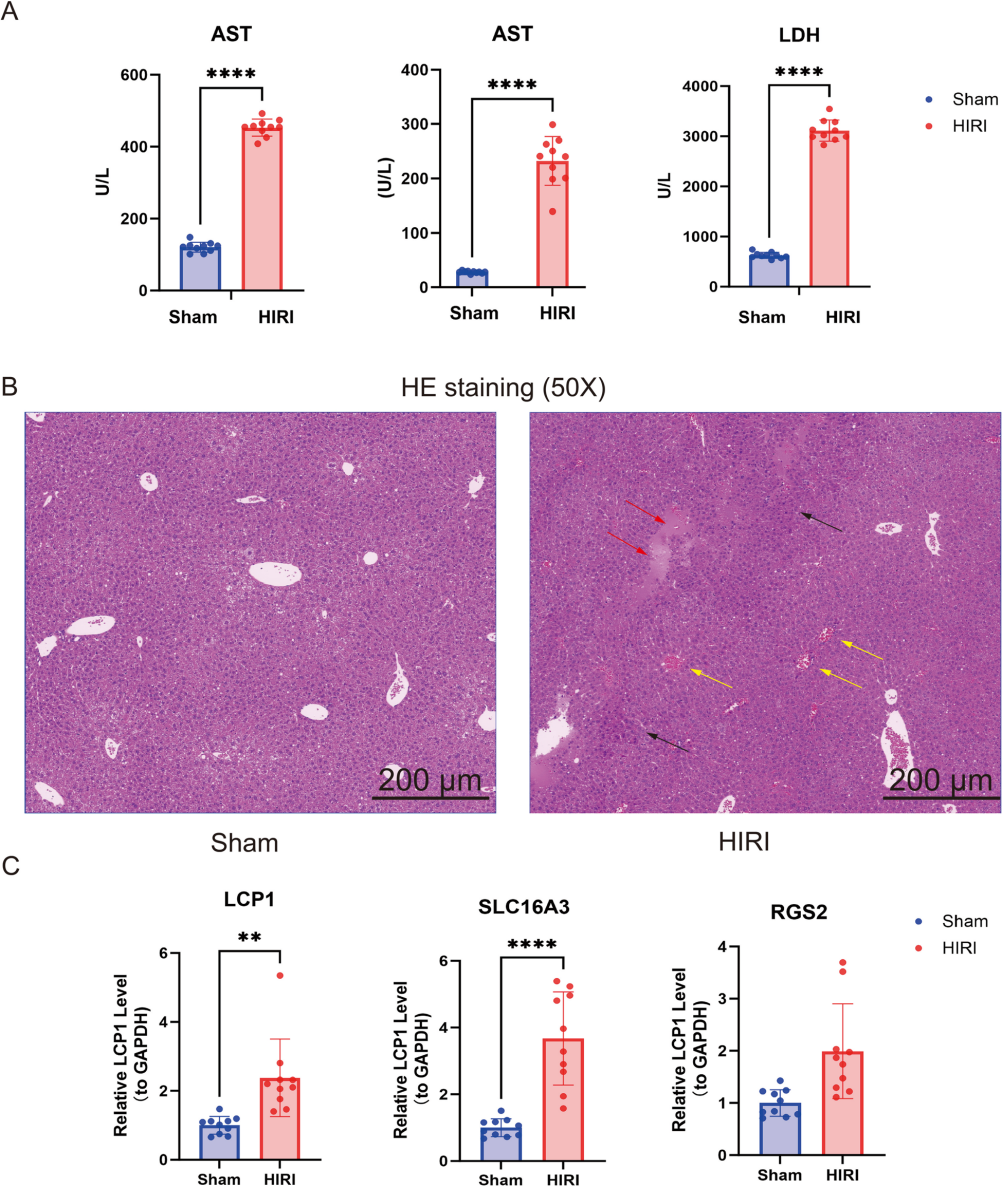
Validation of the HIRI model establishment and increased expression of biomarkers in HIRI samples. **A** ALT, AST, and LDH values in both sham and HIRI mice (n = 10 per group). **B** Representative HE staining of the liver tissue from mice subjected to sham and HIRI. The red arrow points to coagulative necrosis, the black points to inflammatory cell infiltration, and the yellow points to hemorrhage. **C** Bar chart comparing biomarker expression levels between the control group and HIRI group for LCP1, RGS2, SLC16A3. **p < 0.01, ****p < 0.0001

After the successful establishment of the HIRI mouse model, the liver function and histopathology of mice in the two groups were evaluated. Compared with the control group, the HIRI group exhibited significantly impaired liver function, with notably elevated serum levels of ALT, AST, and LDH (Fig. 8A). When analyzing the H&E-stained sections of liver tissues from both groups, the HIRI group displayed more disrupted cellular structures, a greater number of cells with nuclear pyknosis, cytoplasmic hyperchromasia, and shedding necrosis, as well as more pronounced inflammatory cell infiltration than the control group (Fig. 8B). In qPCR experiments, the expression levels of LCP1, SLC16A3, and RGS2 were significantly upregulated in the HIRI samples (*P* < 0.01) (Fig. 8C). These findings are consistent with the expression trends in the training and validation sets, confirming the reliability of the bioinformatics results at the genetic level.

## Discussion

HIRI induces ATP depletion, excessive ROS accumulation, and calcium overload, which collectively trigger mitochondrial dysfunction, oxidative stress, and inflammatory responses [12]. Despite extensive investigation, HSPA12A derived from hepatocytes can alleviate macrophage chemotaxis and inflammatory activation by inhibiting glycolysis-mediated HMGB1 lactic acid modification and secretion, thereby mitigating hepatic ischemia/reperfusion injury [35]. ACD, a newly recognized mode of cell death, is characterized by lysosomal alkalinization, mitochondrial swelling, and impaired autophagic flux [9]. The mechanistic link between ACD and HIRI is increasingly apparent, as ACD may constitute a distinct pathway of hepatocyte and immune cell death contributing to disease progression.

Two HIRI-related transcriptomic datasets, GSE151648 and GSE12720, were extracted from the GEO database. Differential expression analysis identified 586 DEGs, and WGCNA further revealed 762 ACD-associated module genes. Intersection of the two datasets yielded 39 candidate genes, which were refined to 13 through PPI network analysis. Application of two machine learning algorithms, SVM-RFE and random forest (RF), prioritized nine biomarker candidates. Among these, LCP1, SLC16A3, and RGS2 were validated as reliable biomarkers.

Gene enrichment analysis indicated significant associations of the candidate genes with the JAK-STAT and PI3K-Akt signaling pathways. Pharmacological investigations demonstrated that Pemafibrate alleviated HIRI through activation of the JAK2/STAT3–β/PPARα axis [36], whereas Lin28 exerted protective effects by engaging the PI3K-Akt pathway [37]. Despite these observations, the mechanistic basis remains only partially defined. Within the JAK-STAT pathway, STAT3 regulates mitochondrial activity through diverse mechanisms and contributes to apoptosis as well as other programmed cell death processes [38], suggesting that this pathway may participate in mediating cell death during HIRI by regulating the expression of ACD-associated gene signatures. Evidence from myocardial ischemia–reperfusion injury (MIRI) further supports this notion, as activation of PI3K/Akt signaling enhances antioxidant, anti-inflammatory, and autophagic responses while restraining mitochondrial dysfunction and apoptosis [39]. By analogy, in HIRI, this pathway may alleviate mitochondrial damage and exert protective effects by negatively regulating the expression or function of ACD-associated gene signatures.

LCP1, SLC16A3, and RGS2 were identified as biomarkers of ACD in HIRI, each displaying increased expression confirmed by RT-qPCR analysis. LCP1 highly enriched in hematopoietic cells such as lymphocytes, monocytes, and macrophages [40]. Aberrant LCP1 activity has been linked to excessive immune activation and intensified inflammatory responses in autoimmune disorders [41, 42]. During the reperfusion stage of HIRI, pronounced immune infiltration occurs, and LCP1 may aggravate the inflammatory cascade by amplifying immune cell activity [4, 43].

SLC16A3 encodes a monocarboxylate transporter that mediates efflux of lactate, pyruvate, and related substrates, ensuring intracellular pH balance and energy supply [44, 45]. Given that ACD was initially defined as a specific form of cell death in CD8⁺T cells, the overexpression of SLC16A3 may influence ammonia metabolism by regulating intracellular pH, thereby promoting ACD occurrence and leading to T cell dysfunction. This mechanism could partially explain the immune dysregulation observed in HIRI. On the other hand, recent studies indicate that SLC16A3 also participates in regulating ferroptosis: inhibiting its expression can reduce extracellular lactic acid accumulation, ameliorate the hypoxic tumor microenvironment, and induce ferroptosis in HCC by blocking ERK pathway activation [46]. These findings suggest that SLC16A3 may play distinct roles in different types of cell death. To clarify its mechanism, follow-up studies need to verify its phenotypic effects on ammonia-induced cell death through overexpression or knockdown of SLC16A3, systematically elucidating the specific mechanism of SLC16A3 in ACD and its pathological significance in the HIRI process.

These three genes were significantly enriched in the pathways of "ribosome," "oxidative phosphorylation," and "complement and coagulation cascades." Enrichment in the ribosome pathway suggests these genes may influence protein synthesis, cell growth, and repair processes [47]. During hepatic ischemia, tissue hypoxia severely impairs mitochondrial function, particularly disrupting oxidative phosphorylation. This leads to a significant reduction in intracellular ATP production and an accumulation of ROS [48]. ROS not only cause direct cellular damage but also activate inflammatory pathways, such as promoting the release of inflammatory factors from Kupffer cells, thereby amplifying the inflammatory response [49]. The complement system, a key component of innate immunity, can mediate inflammatory responses upon activation [50]. Aberrant activation of the complement and coagulation cascades can exacerbate inflammation, disrupt the blood–brain barrier, and cause abnormal synaptic pruning, among other detrimental effects [51]. Notably, ammonia-induced cell death is closely associated with inflammation [52], which may further activate the complement and coagulation pathways [53], creating a vicious cycle that aggravates cellular injury. In summary, these three genes may collectively drive the progression of HIRI by modulating ribosomal function, energy metabolism, and the complement–coagulation network, thereby influencing critical processes like oxidative stress and inflammation.

Combined analysis of immune infiltration and single-cell sequencing reveals that mononuclear phagocytes, such as macrophages and immature dendritic cells, play a crucial role in HIRI. This finding suggests that the progression of HIRI may be closely associated with the activation of the ACD pathway in mononuclear phagocytes. Existing studies have indicated that the polarization of macrophages toward the M1 phenotype is one of the key factors exacerbating HIRI. During HIRI, the excessive production of mitochondrial ROS not only directly damages hepatocytes but also further promotes the M1 polarization of macrophages, thereby driving the inflammatory cascade [54]. Furthermore, as a key ammonia detoxifying enzyme, the deficiency of glutamine synthetase significantly promotes the activation of monocyte-derived macrophages. In contrast, inhibiting this activation by neutralizing CCL2 can effectively alleviate acute liver injury [55], which further establishes a clear intrinsic link between ammonia metabolism disorders and immune activation. Notably, single-cell analysis results show that hepatocytes account for a small proportion in both HIRI and normal groups. Moreover, there is no significant difference in the expression of three ACD-related biomarkers (LCP1, SLC16A3, RGS2) in hepatocytes. This implies that the ammonia-induced cell death process may be more likely to occur in mononuclear phagocytes. On the other hand, it is also possible that hepatocytes may be partially lost due to their fragility during sample preparation, which affects their representativeness in the analysis and may mask potential subtle differences in expression. Therefore, future studies need to further optimize the sample preparation process and utilize more sophisticated cell sorting techniques to accurately elucidate the specific mechanism of the ACD pathway in hepatocytes and various immune cells during HIRI.

This investigation represents the first effort to examine gene signatures associated with ACD in HIRI, identifying LCP1, SLC16A3, and RGS2 as novel biomarkers and establishing a foundation for elucidating their regulatory functions in HIRI pathogenesis. Nonetheless, several limitations warrant consideration. First, reliance on sequencing data from public databases precludes direct confirmation of ACD occurrence in HIRI or precise delineation of its role. Future research should incorporate quantification of ammonia concentrations in liver tissue or serum, alongside the application of fluorescent probes such as LysoTracker and JC-1 to detect lysosomal alkalinization and mitochondrial dysfunction, thereby clarifying the mechanistic development of ACD in HIRI. Second, this study validated expression alterations of the three genes solely at the genetic level; subsequent investigations should employ gene overexpression or knockdown strategies to assess corresponding protein expression changes and elucidate their regulatory roles within the ACD pathway through functional assays. Third, the current nomogram prediction model, based on gene expression profiles, exhibits limited clinical applicability and requires refinement through integration of additional clinicopathological variables and validation in large-scale, multicenter cohorts. Furthermore, due to the nascent understanding of ACD, single-cell RNA sequencing technology was not utilized in this study. Future work could leverage scRNA-seq to analyze expression patterns of ammonia death-related markers within hepatocyte subpopulations and investigate whether immune cells demonstrate analogous response profiles, thereby enabling comprehensive construction of a cell-type-specific regulatory network of ACD in HIRI. Collectively, the results suggest a mechanistic involvement of ACD-related genes signatures in the progression of HIRI and provide new avenues for diagnostic development, therapeutic strategies, and drug discovery.

## Conclusions

LCP1, SLC16A3, and RGS2 have been identified as biomarkers of HIRI. The study advances understanding of ACD-related genes signatures in HIRI and provides a foundation for future mechanistic research and therapeutic development.

## Supplementary Information

Additional file 1. Information on 467 ammonia-induced death-related genes

Additional file 2. Reverse transcription reaction program

Additional file 3. Amplification conditions

Additional file 4. Information on 762 key module genes

Additional file 5. Information on 586 DEGs

Additional file 6. GO enrichment results for candidate genes

Additional file 7. The nomogram was able to predict the risk of HIRI in samples. **A** Nomogram model of diagnostic characteristics for biomarkers. **B** Calibration curve of the nomogram model. **C** DCA curve of the nomogram model. **D** ROC curve of the nomogram prediction performance.

Additional file 8. KEGG enrichment results for LCP1

Additional file 9. KEGG enrichment results for RGS2

Additional file 10. KEGG enrichment results for SLC16A3

Additional file 11. Comparison of infiltration levels across 64 immune cell types between HIRI samples and normal samples using the xCell method. **A** Heatmap of immune scores for 64 immune cell types between HIRI and control groups. **B** Differentially expressed immune cells between HIRI and control groups. **C** Heatmap of biomarker correlations for differentially expressed immune cells. *p < 0.05, **p < 0.01, ***p < 0.001.

Additional file 12. Data processing and dimensionality reduction clustering. **A** Before quality control. **B** After quality control. **C** Screening for highly variable genes. **D**–**E** PCA analysis results for single-cell datasets.

## Data Availability

The dataset(s) supporting the conclusions of this article is(are) available in the [GEO] at [https://www.ncbi.nlm.nih.gov/gds] with accession numbers (GSE151648，GSE171539 and GSE12720), [MSigDB] at [https://www.gsea-msigdb.org/gsea/msigdb].

## Abbreviations

IRI: Ischemia–reperfusion injury
HIRI: Hepatic ischemia–reperfusion injury
ROS: Reactive oxygen species
ACD: Ammonia-induced cell death
RHCG: Rh family C glycoprotein
TCA: Tricarboxylic acid
ACDRGs: AD-related genes
WGCNA: Weighted gene co-expression network analysis
PPI: Protein–protein interaction
GSEA: Gene set enrichment analysis
GEO: Gene expression omnibus
IRI⁺: HIRI cases
IRI⁻: Control hepatic tissue samples
DEGs: Differentially expressed genes
GO: Gene ontology
KEGG: Kyoto encyclopedia of genes and genomes
BP: Biological process
CC: Cellular component
MF: Molecular function
SVM-RFE: Support vector machine–recursive feature elimination
DCA: Decision curve analysis
ROC: Receiver operating characteristic
AUC: Area under the curve
miRNAs: MicroRNAs
lncRNAs: Long non-coding RNAs
FDR: False discovery rate
NES: Normalized enrichment score
GO-BP: Gene ontology-biological process
GO-CC: Cellular component
GO-MF: Molecular function
GGI: Gene–gene interaction
MDSCs: Myeloid-derived suppressor cells
RF: Random forest
MIRI: Myocardial ischemia–reperfusion injury
NK: Natural killer
DCs: Dendritic cells
ALT: Alanine aminotransferase
AST: Aspartate aminotransferase
LDH: Lactate dehydrogenase
