# Supplementary figures and images for: Transcriptomic exploration combined with experimental validation: uncovering the potential value of biomarkers related to ammonia-induced cell death in hepatic ischemia–reperfusion injury
Source: Eur J Med Res. 2025 Nov 26;30:1180. doi: 10.1186/s40001-025-03450-1 (PMC12659301; doi:10.1186/s40001-025-03450-1)

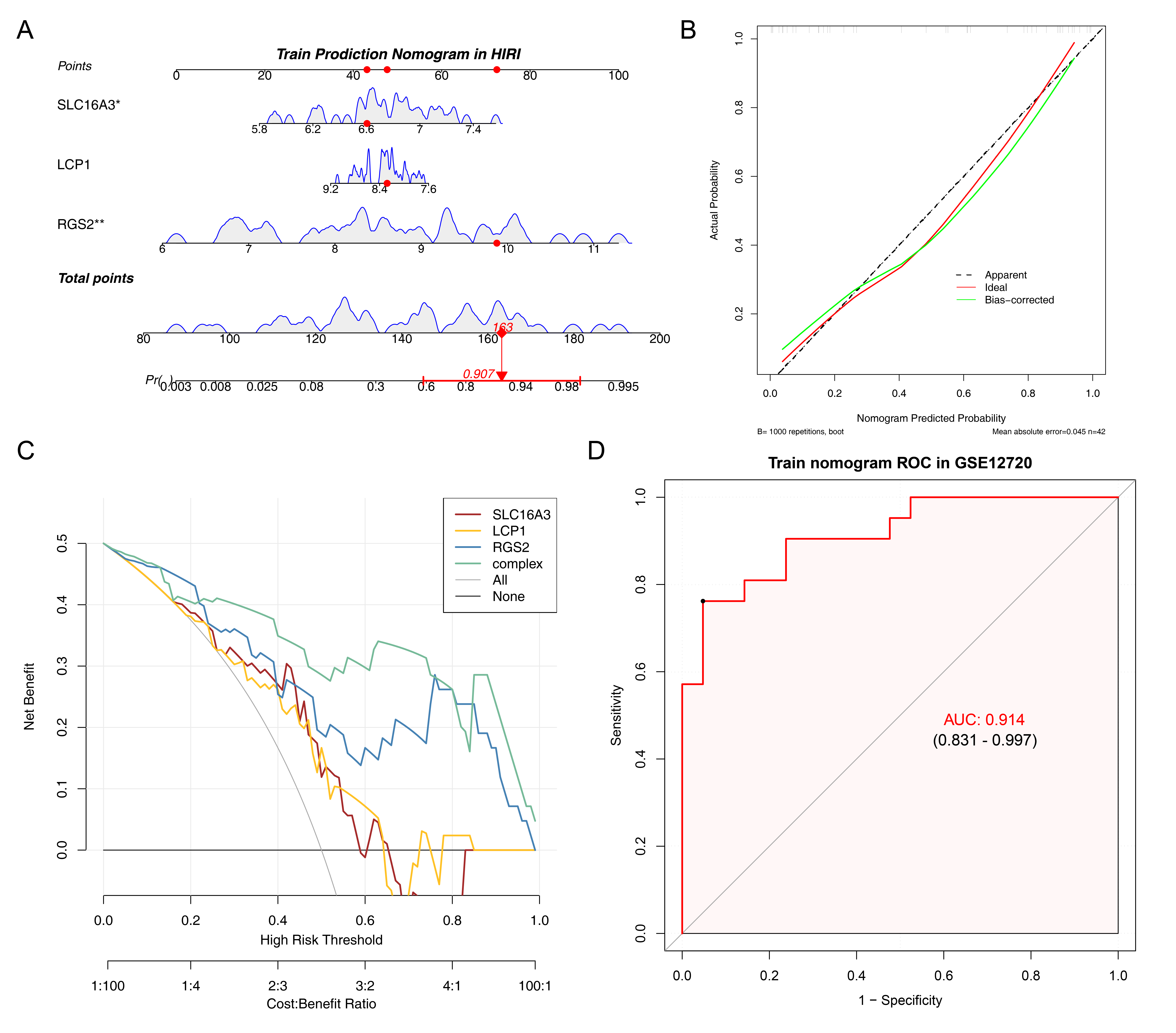

Supplement: Supplementary file 7 — Additional file 7. The nomogram was able to predict the risk of HIRI in samples. A Nomogram model of diagnostic characteristics for biomarkers. B Calibration curve of the nomogram model. C DCA curve of the nomogram model. D ROC curve of the nomogram prediction performance. [file 40001_2025_3450_MOESM7_ESM.tif]

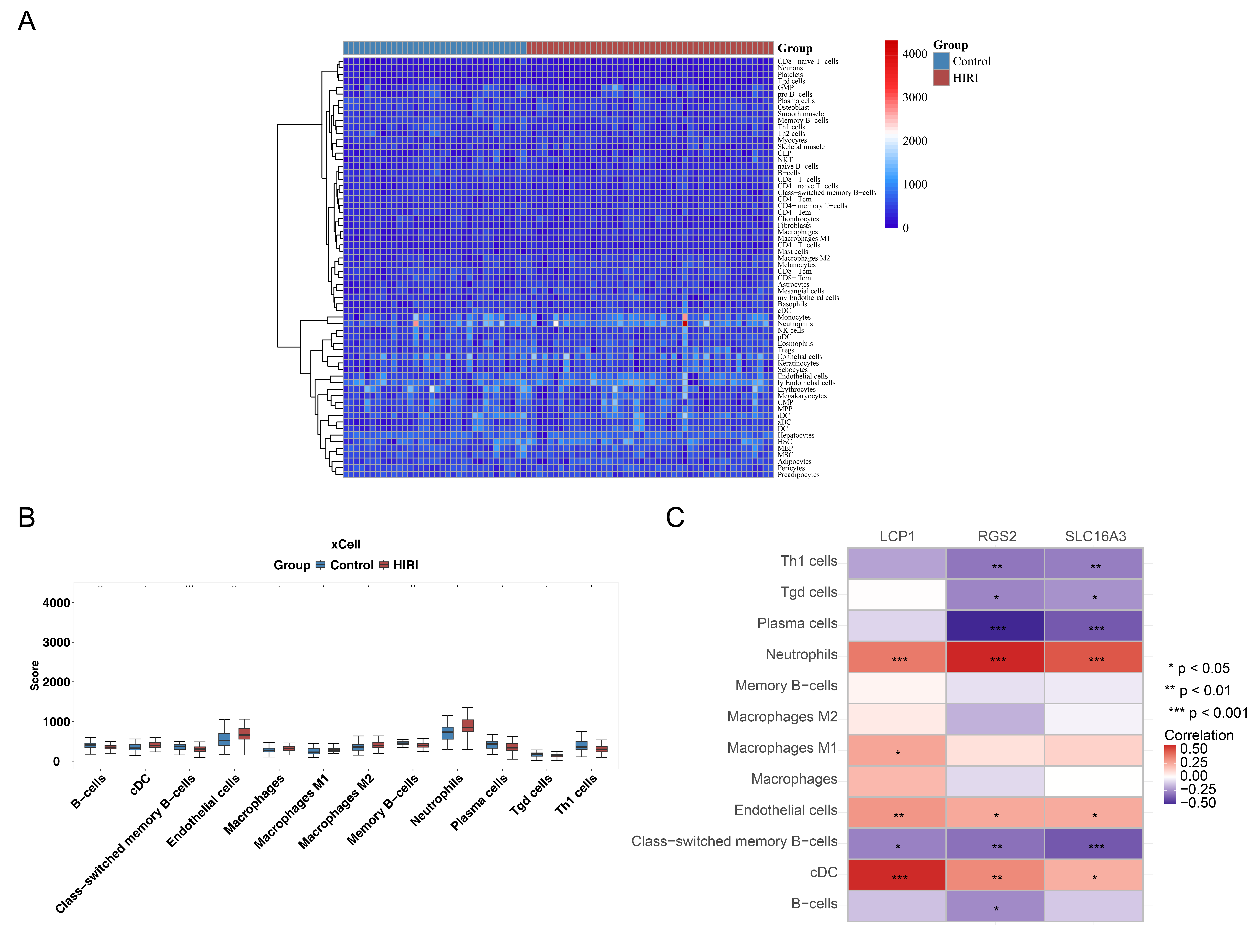

Supplement: Supplementary file 11 — Additional file 11. Comparison of infiltration levels across 64 immune cell types between HIRI samples and normal samples using the xCell method. A Heatmap of immune scores for 64 immune cell types between HIRI and control groups. B Differentially expressed immune cells between HIRI and control groups. C Heatmap of biomarker correlations for differentially expressed immune cells. *p < 0.05, **p < 0.01, ***p < 0.001. [file 40001_2025_3450_MOESM11_ESM.tif]
